# Clinical Proteomics of the Neglected Human Malarial Parasite *Plasmodium vivax*

**DOI:** 10.1371/journal.pone.0026623

**Published:** 2011-10-20

**Authors:** Pragyan Acharya, Rani Pallavi, Syama Chandran, Vrushali Dandavate, Syed Khund Sayeed, Ankit Rochani, Jyoti Acharya, Sheetal Middha, Sanjay Kochar, Dhanpat Kochar, Susanta Kumar Ghosh, Utpal Tatu

**Affiliations:** 1 Department of Biochemistry, Indian Institute of Science, Bangalore, Karnataka, India; 2 Department of Medicine, S. P. Medical College, C-54, Sadul Ganj, Bikaner, Rajasthan, India; 3 National Institute of Malaria Research (ICMR Complex), Devanahalli, Bangalore, India; Institut National de la Santé et de la Recherche Médicale - Institut Cochin, France

## Abstract

Recent reports highlight the severity and the morbidity of disease caused by the long neglected malaria parasite *Plasmodium vivax*. Due to inherent difficulties in the laboratory-propagation of *P. vivax*, the biology of this parasite has not been adequately explored. While the proteome of *P. falciparum*, the causative agent of cerebral malaria, has been extensively explored from several sources, there is limited information on the proteome of *P. vivax*. We have, for the first time, examined the proteome of *P. vivax* isolated directly from patients without adaptation to laboratory conditions. We have identified 153 proteins from clinical *P. vivax*, majority of which do not show homology to any previously known gene products. We also report 29 new proteins that were found to be expressed in *P. vivax* for the first time. In addition, several proteins previously implicated as anti-malarial targets, were also found in our analysis. Most importantly, we found several unique proteins expressed by *P. vivax*.This study is an important step in providing insight into physiology of the parasite under clinical settings.

## Introduction

*Plasmodium vivax* has been long neglected as a major malaria parasite since it is the causative agent of benign malaria, in favor of *Plasmodium falciparum* that causes fatal cerebral malaria. However, *P. vivax* is the second leading cause of malaria outside Africa inflicting about 40% of the world population. In Asia, *P. vivax* accounts for about 50% of malaria cases [1]. *P. vivax* is known to cause severe morbidity in individuals infected with this parasite leading to paroxysmic fever, severe anemia and thrombocytopenia [1]. Although drug-resistant species of both *P. vivax* as well as *P. falciparum* have been reported, *P. vivax* vaccine and drug target discovery operate only in the wake of *P. falciparum* vaccine and drug target development [2].

Evidences from recent studies have suggested significant differences in the gene families employed by these parasites. Genome analysis of *P. vivax* isolated from malaria patients have shown that in spite of its resemblance with other malaria parasites in its gene content and metabolic processes, *P. vivax* possesses novel gene families and alternative invasion pathways [3]. These differences may lead to distinctive clinical features of *P. vivax*. For example, unlike *P. falciparum* which infects red blood cells, *P. vivax* preferentially infects reticulocytes expressing Duffy blood group antigens (Fya and Fyb), which act as receptors for proteins PvRBP1 and PvRBP2 expressed at the apical pole of *P. vivax* merozoites. Additionally, the presence of hypnozoite stage and the absence of cytoadherence in *P.vivax* make its biology different from that of *P. falciparum*. Due to these differences in the biology as well as clinical properties of *P. vivax* and *P. falciparum*, our knowledge of *P. falciparum* cannot be extrapolated to *P. vivax*.

One of the major hurdles in the study of *P. vivax*-malaria is the absence of a long-term *in vitro* culture system, unlike *P. falciparum* [4]. As a result, our knowledge about *P. vivax* is limited to a few studies that have been carried out using short-term cultures of *P. vivax* and clinically isolated parasites. Our current understanding about the *P. vivax* life cycle is restricted only to the information provided by the global transcriptome analyses of this parasite from clinical samples and limited proteomics analysis of only schizont stage *P. vivax* parasites that have been culture adapted [5], [6], [7]. Although transcriptome analysis provides useful understanding at the level of gene expression, they do not reflect the active protein component of a cell. Further, parasites such as *Plasmodium*, interact with and respond to host environmental cues which can often be revealed by proteomic analyses of the parasite as it is present within the host. However unlike *P. falciparum*, the proteome of this parasite has not yet been explored either from short-term culture adapted parasites or parasites directly isolated from malaria patients. Dearth of any available information regarding the active protein profile employed by this parasite in order to invade and establish infection in humans makes it imperative to carry out proteomic analysis of the parasite directly isolated from patients who suffer from vivax-malaria. This will indeed facilitate deeper understanding of the pathology of this disease. The proteomic analysis of *P. vivax*, however, is hampered by limited parasitemia of usually <0.5%, seen in case of *P. vivax* infections as it infects only reticulocytes, which comprise 1–3% of the total RBCs [8]. This is in contrast to *P. falciparum* which is able to infect RBCs of all ages, resulting in a relatively higher average parasitemia of >5%.

Our previous study [9] gave the first ever insight into the proteome of malarial parasites at the clinical level. In the case of *P. falciparum* a reference proteome from lab cultures existed prior to the analysis of the clinical proteome [10], [11]. However, knowledge of *P. vivax* proteome is extremely limited [7]. In this study, we report the first ever in-depth proteomic analysis of *P. vivax* isolated directly from malaria patients. We were able to identify 153 proteins from the blood stages of *P. vivax*. Interestingly, more than 36% of the parasite proteome comprised of hypothetical proteins. We were also able to identify 16 proteins for which there are no homologs in *P. falciparum*. Overall, our analysis reinforces the belief that *P. vivax* biology needs to be examined independent of *P. falciparum* and has several novel features unique to this organism that can be exploited for therapeutic intervention against this major parasite.

## Materials and Methods

### Ethics Statement

The present study has been approved by the Institutional Scientific Advisory Committee of National Institute of Malaria Research (ICMR), New Delhi, India. A written informed consent was obtained from each patient participated in this study.

### Collection of Parasites from Patients

5 ml of blood was collected in heparin coated tubes from patients diagnosed with malaria (with written informed consent). Microscopic examination of peripheral blood smear was done for the presence of malarial parasites. Additionally, Falcivax rapid diagnostic test based on PfHRP-2 and *P. vivax* specific LDH was used for the differentiation of *P. falciparum* and *P. vivax* malaria. Samples positive only for *P. vivax* were used for this study. Here we must mention that RDT may sometimes detect mixed infections of *P. vivax* with *P. falciparum*. In order to avoid this, more specific tests such as nested PCR can be performed. However, since samples used in our study were directly taken from patients for proteomics-processing, we have ensured greater stringency during data analyses as detailed below.

### Sample Preparation

Parasitized whole blood cells were centrifuged at 1,500 g for 15 min for the removal of plasma and buffy coat. Blood pellet thus obtained was diluted with PBS and was layered on Histopaque (Sigma Aldrich) for the removal of white blood cells. *P. vivax* infected cells was purified from uninfected cells by layering the cell suspension on a 45, 50 and 65% Percoll gradient. An enriched fraction of asexual stages of *P. vivax* was obtained with little contamination of host cells. For extraction of *P. vivax* proteins, enriched parasitized cells were either boiled directly in SDS sample buffer or lysed with Saponin (Fluka) to obtained soluble and insoluble fractions. Saponin insoluble pellets were further processed as described earlier [12]. Briefly, the saponin pellet was lysed first in Triton X containing buffer, Buffer A (10 mM Tris HCl, pH 7.4; 5 mM EDTA; 1% Triton X 100) followed by lysis in urea containing buffer, Buffer B (10 Mm Tris HCl, pH 7.4; 5 mM EDTA; 1% SDS; 6 M Urea). Soluble fractions obtained at each step were acetone precipitated and the final insoluble pellet was directly boiled in SDS loading buffer. Purified parasites or saponin pellet was maintained at 4°C throughout the lysis procedure. All theses samples were separated on a 10% SDS-PAGE followed by in gel digestion as described by Wilm et al. 1996 with slight modification [13]. In brief, each lane was cut into pieces of width 2 mm. These pieces were further chopped into small pieces, which were reduced with 10 mM dithiotreitol followed by alkylation using 55 mM iodoacetamide. Alkylated gel pieces were incubated with 400 ng of trypsin (20 µl of trypsin from the solution of 20 ng/µl trypsin (Trypsin Gold, Promega), in 25 mM NH_4_OAc for 14–16 hr at 37°C. Trypsin digested peptides were eluted from the gel pieces using 60% acetonitrile (ACN) and 5% formic acid and were subjected to LC-MS/MS.

### Nano LC-MS/MS analysis of malaria proteins

Digested peptides were analyzed by nano LC- MS/MS as described previously [8]. Briefly, peptide mixtures were dissolved in 25 µl of sample preparation solution (98% water, 2% Acetonitrile and 0.5% Formic acid) and injected into Nano-LC through an autosampler system. Peptides were eluted using nano- reverse phase column (Michrom C18 5 µ 300 Å) which was further connected to the Nano Spray ESI- QTOF system (Qstar Elite, Applied Biosystems). A gradient of water and acetonitrile was set up for 60 minutes with a flow rate of 400 nL/minute. Eluted peptides from the column were ionized using ESI source with ion spray voltage 2250 V and temperature 120°C. Ionized peptides were analyzed by one full MS scan and four consecutive product ion scans of the four most intense peaks, using rolling collision energy. An Information Dependant Acquisition (IDA) experiment was used to specify the criteria for selecting each parent ion for fragmentation which included selection of ions in m/z range: >400 and <1600, of charge state of +2 to +5, exclusion of former target ions for 30 seconds, accumulation time of 1 second for a full scan and 2 seconds for MS/MS. The data generated by the Analyst software was stored in a .wiff format.

### Data Analysis

Data was analyzed using ProteinPilot version 4.0 software. The original data files were analyzed using the ProteinPilot version 4.0 software with a combined database (NCBI Human DB 2008 (containing 39125 non-redundant protein entries, 18.8 Mb), PlasmoDB *Plasmodium vivax* version 7.1 (containing 5432 redundant protein entries, 4.4 Mb), and PlasmoDB *Plasmodium falciparum* October 2008 (containing 11669 redundant protein entries, 4.8 Mb) with a total of 56226 protein entries using Paragon Algorithm. The number of missed cleavages permitted was two. During the analysis, in the search parameters modification of cysteine by idoacetamide and biological modifications programmed in algorithm were allowed. Mass tolerance for precursor ion and fragment ions were set to 100 ppm and 0.2 Da respectively. In Paragon Algorithm, protein score is calculated on the basis of percentage confidence level of the peptides identified. Protein score of minimum 0.47 (fit incorrect rate is 0%) corresponding to a confidence level greater than 66% were used. Several *P. vivax* proteins detected had a score of less than 0.47 and these have been included in a separate list in Table S6.

In order to rule out false discoveries, we have carried out False Discovery Rate (FDR) analysis. For this, ProteinPilot 4.0 with Paragon algorithm has been used for data analysis. As part of the Paragon analysis method, a false discovery rate (FDR) analysis of the results has been carried out by the Proteomics Performance Evaluation Pipeline Software (PSPEP). Finally, proteins have been selected on the basis of their critical FDR value i.e. 1%.

In order to avoid identifications based on redundant peptides in our proteome, we have not included proteins that have no unique peptide identifications (Table S4). Proteins which share some peptides as well as have unique peptide-identifications, have been grouped accordingly.

### Annotation of hypothetical proteins

About 36% of the *P. vivax* proteome consisted of hypothetical proteins. We have carried out sequence based domain search using the SMART and Pfam tools and identifying domains present in them. A molecular class was assigned to the hypothetical proteins based on the identified domain.

## Results

### Functional classification of proteins expressed by *P. vivax*

**Figure 1:**
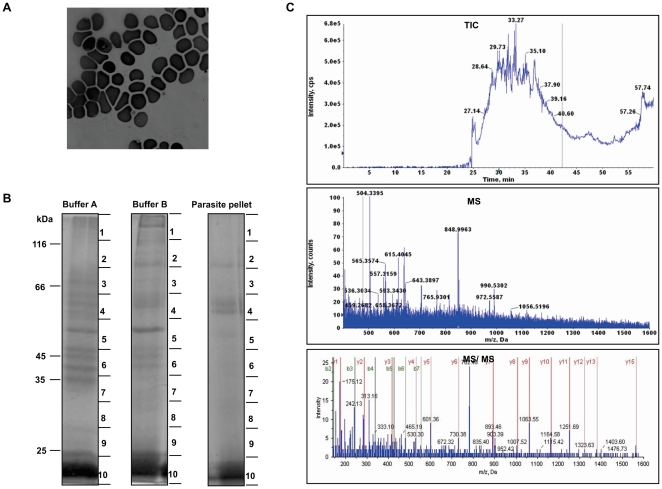
Mass spectrometric analysis of proteins employed by asexual stages of *P. vivax*. A. Giemsa-stained image of peripheral blood smear of *P. vivax* infected patient. B. SDS-PAGE profile of proteins extracted from asexual stages of *P. vivax* by sequential lysis using SDS buffer (lane 1), urea containing buffer (lane 2) and direct boiling of pellet in Laemmli buffer (lane 3). C. Represents the Total Ion Chromatogram (TIC), MS, and MS/MS spectra for enolase (PVX_095015).

**Figure 2:**
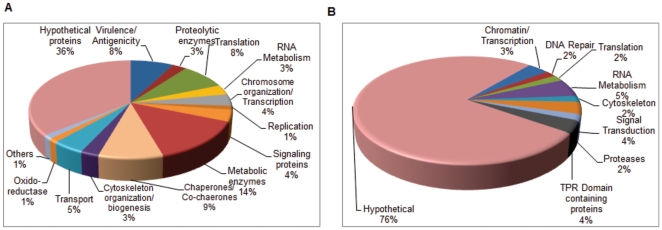
Functional profiles of protein expressed by asexual stages of *P. vivax*. A. Proteins identified in asexual stages were plotted as a function of their broad functional classification as defined in PlasmoDB or GO. Only one class was assigned to one protein to avoid any redundancy. Major group in the *P. vivax* is represented by hypothetical proteins followed by metabolic enzymes, chaperones and proteins involved in virulence. B. Showing functional plot for hypothetical proteins. About 25% of the hypothetical proteins were assigned to different functional classes on domain architecture.

To study the proteome of *P. vivax*, we collected peripheral blood from *P. vivax* infected patients attended to the OPD of Wenlock Government Hospital, Mangalore, Karnataka, India and S.P. Medical College and Associated Group of Hospitals, Bikaner, India. *P. vivax* infected blood was collected before administration of any drugs. To minimize the host proteins contamination parasitized erythrocytes were purified by percoll density gradient as described in “Materials and Methods”. A typical sample consisting of about 5 mL of parasitized blood of 0.1% parasitemia contains 1×10^7^ infected reticulocytes. However, at each step of purification there is a loss of sample which therefore, resulted in a 1×10^5^ infected reticulocytes after percoll purification. Proteins were extracted from enriched fraction either by directly boiling in sample buffer or by sequential lysis using SDS containing buffer or urea containing buffer. After each lysis step proteins were separated on SDS-PAGE (Figure 1B). Each of these lanes were divided into 10 pieces and were subjected to in-gel trypsin digestion. Digested peptides were extracted in 60% ACN and 5% formic acid and were analyzed by LC-MS/MS-QTOF. The representative TIC, MS and MS/MS spectra for one of the sample is given in Figure 1C. We were able to identify 137 proteins from the asexual stages of *P.vivax*. In our previous study, we have identified 16 *P. vivax* proteins. Altogether, we here represent 153 proteins expressed by asexual stages of *P. vivax* (Table S1). *P. vivax* proteome comprises of proteins consisting of chaperones, glycolytic enzymes, signaling proteins, RNA processing, chromatin and chromosome organization, cytoskeleton organization, transport, virulence, transcription and translational regulatory and hypothetical proteins (Figure 2A). Majority of these proteins belong to the hypothetical protein group followed by proteins involved in metabolic processes, protein folding (chaperones and co-chaperones) and pathogenicity. Based on the presence of domains, we have classified hypothetical proteins into various categories along with other annotated proteins as described below. Of all the hypothetical proteins about 76% did not contain any identifiable domains. From here onwards, the annotated hypothetical proteins are no longer analyzed as hypothetical proteins. Instead, they are categorized as proteins with known putative functions (Figure 2B) (Table S2).

Additionally, we have identified 7 proteins which were entirely specific to *P. vivax* (do not contain homologs in any *Plasmodium* species) and 16 proteins for which there were no homologs in *P. falciparum* (Table 1). Of the 16 proteins that did not have homologs in *P. falciparum*, 2 were Vir proteins and 8 were P-fam proteins, known to be involved in virulence/antigenicity of *P. vivax*.

**Table 1:** List of uniquely expressed proteins in *P. vivax* isolated from patients.

Serial No.	Accession No.	Protein	Mw/pI	Probable function
1	PVX_074690*	Variable surface protein Vir4-related	48.4/9.29	Virulence/Antigenicity
2	PVX_096985*	Variable surface protein Vir, putative	44.6/5.88	Virulence/Antigenicity
3	PVX_101520*	Pv-fam-d protein	49.5/9.7	Virulence/Antigenicity
4	PVX_003525*	Hypothetical protein	211.6/4.49	Not Known
5	PVX_089835*	RAD protein	398.77/10.01	Virulence/Antigenicity
6	PVX_092995*	Tryptophan-rich antigen (Pv-fam-a)	429.99./10.1	Virulence/Antigenicity
7	PVX_118695*	Pv-fam-d protein	103.2/9.85	Not Known
8	PVX_003545	Hypothetical protein, conserved	34.4/6.31	Not Known
9	PVX_003555	Hypothetical protein, conserved	119.2/3.82	Not Known
10	PVX_083555	Hypothetical protein	16.5/4	Not Known
11	PVX_090265	Tryptophan-rich antigen (Pv-fam-a)	40.1/6.57	Virulence/Antigenicity
12	PVX_096950	Tryptophan-rich antigen (Pv-fam-a)	39.8/9.92	Virulence/Antigenicity
13	PVX_092990	Tryptophan-rich antigen (Pv-fam-a)	157.5/3.36	Virulence/Antigenicity
14	PVX_112670	Tryptophan-rich antigen (Pv-fam-a)	38.5/6.11	Virulence/Antigenicity
15	PVX_090230	Early transcribed membrane protein (ETRAMP)	15.8/10.78	Virulence/Antigenicity
16	PVX_097880	Hypothetical protein	145/10.33	Not Known

‘*’Refers to proteins only present in *P. vivax*. Remaining proteins are present in any one species of *Plasmodium* except *P. falciparum*.

### Drug targets and vaccine candidates in *P. vivax*

Several proteins that are well known drug targets and vaccine candidates in *P. falciparum* are also expressed in *P. vivax* (Table 2). Plasmepsin IV, m1-family aminopeptidaese, spermidine synthase, fructose 1, 6- bisphosphate aldolase, pyridoxal kinase, triosephosphate isomerase, L-lactate dehydrogenase, Hsp90 and Hsp70 are all well known drug targets in *P. falciparum* against which inhibitors are known [14]–[28]. Some vaccine candidates such as the duffy receptor precursor, which is unique to *P. vivax*, were also detected [3], [29], [30]. Earlier, sequencing of *P. vivax* revealed several novel gene families which are located in the subtelomeric regions [3]. Most notably, we found at least five members of Pv-fam-a (tryptophan rich antigen) in patients suffering from *vivax* malaria (Table 2). There are at least 36 members of pv-fam-a family of proteins, one of which has previously been reported to elicit humoral immune response and hence a probable vaccine candidates [31]. Table 2 contains a list of potential drug targets/vaccine candidates in *P. vivax* whose efficacy can be revealed only upon further research.

**Table 2:** Putative vaccine candidates and drug targets identified in *P. vivax* isolated from patients.

Potential Drug/Vaccine Targets		Putative Function	Drug Inhibitors (If known)	Ref.
Protein ID	Description			
PVX_092990 PVX_092995 PVX_112670 PVX_096950 PVX_090265	tryptophan-rich antigen (Pv-fam-a)	Immune evasion	-	[3], [30]
PVX_093680	Phist protein (Pf-fam-b)	Immune evasion		[3]
PVX_118695 PVX_101520	Pv-fam-d protein	Immune evasion	-	[3]
PVX_089835	RAD protein (Pv-fam-e)	Immune evasion	-	[3]
PVX_110810	Duffy receptor precursor	Invasion	-	[28]
PVX_086040	Aspartic Protease Plasmepsin IV	Hemoglobin degradation	*C*_2_-symmetric compounds encompassing the 1,2-dihydroxyethylene scaffold and a variety of elongated P1/P1′ side chains	[13]
PVX_122425	M1-family aminopeptidase, putative	hemoglobin digestion	hPheP[CH2]Phe, Co4, and Bestatin	[14], [15]
PVX_092065	Spermidine synthase, putative	Spermine synthesis	cyclohexylamine, dicyclohexylamine	[16]
PVX_118255	Fructose 1,6-bisphosphate aldolase, putative	Glycolysis and Invasion	*-*	[17]–[19]
PVX_113935	Pyridoxal kinase	Biotin Synthesis	6-diazo-5-oxo-L-norleucine	[20]
PVX_118495	Triosephosphate isomerase, putative	Glycolysis	Synthetic interface peptides	[21], [22]
PVX_116630	Lactate dehydrogenase	Glycolysis	Gossypol derivatives, oxamate derivatives	[23]
PVX_087950	Hsp90	Signal transduction, Cell proliferation	*Geldanamycin*, 17AAG	[24], [25]
PVX_089425	Hsp70	Chaperone activity	*pyrimidinone-amides*	[26], [27]

### Comparison of *P. vivax* proteome with transcriptome

All studies on *P. vivax* till date have been carried out with either parasites directly isolated from malaria patients or with isolates adapted to short term culture. The amount of information available about the gene expression and protein expression profiles of *P. vivax* is limited to two transcriptome studies [5], [6] and this proteomics study. Upon closer examination/inspection of proteins reported in this study with two transcriptome datasets we found no transcript evidence for three proteins. These are PVX_114832 (Elongation factor 1 alpha, putative), PVX_250300 (ADP/ATP transporter on adenylate translocase, putative) and PVX_220290 (putative cyclophilin). This observation is intriguing as the previous two studies have used parasite materials from two different geographically distinct regions (one from Thailand and the other from Peru) [5], [6]. Furthermore, these studies included the stage specific transcriptome of intraerythrocytic developmental cycle. Individual differences in gene expression profiles among field isolates have been proposed to reflect parasites adaptability in response to host environment [32], [33]. Based on proteomic evidence from the field isolates of *P. vivax*, we propose that it is possible that these three genes, for which no transcript data exists, either reflect an altered physiological state or are expressed specifically in Indian isolates. This interpretation is based on the assumption that probes for all *P. vivax* genes have been included in the transcriptome analysis.

In addition, we found 14 proteins which show differential expression at transcript levels in different individuals from the two transcriptome studies mentioned above, which may relate to different physiological states [5], [6]. These are PVX_122850 (Dihydrolipoyllysine-residue succinyltransferase component of 2-oxoglutarate dehydrogenase complex, putative), PVX_099275 (hypothetical protein), PVX_080555 (hypothetical protein), PVX_086990 (vacuolar ATP synthase subunit E), PVX_095195 (ATP-dependent RNA helicase, putative), PVX_099095 (elongation factor 1B, putative), PVX_121930 (hypothetical protein), PVX_092630 (hypothetical protein), PVX_098620 (hypothetical protein), PVX_124190 (hypothetical protein), PVX_113635 (hypothetical protein), PVX_101480 (hypothetical protein), PVX_087690 (hypothetical protein) and PVX_110810 (Duffy receptor precursor). As is evident, nine of these are annotated as hypothetical in PlasmoDB database. Domain analysis of these hypothetical proteins revealed that two of them have DNA repair (PVX_099275) and chromatin assembly (PVX_092630) as a putative function. These could be proteins involved in responding to environmental cues and modulating pathways that may affect parasite survival.

### The *P. vivax* interactome

**Figure 3:**
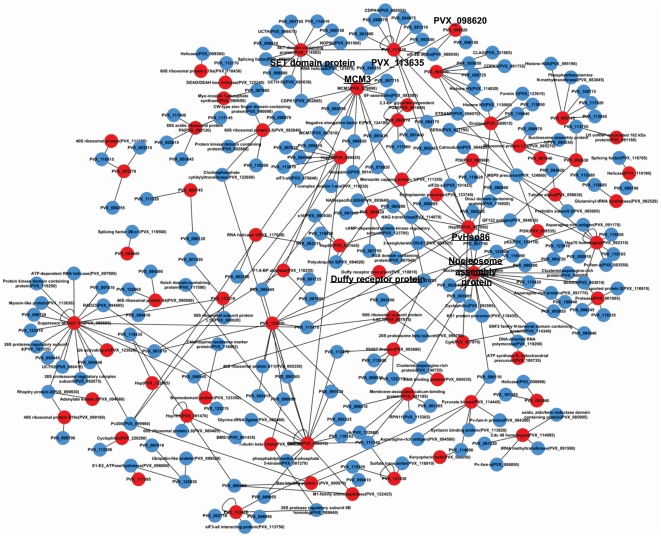
Interaction network of the proteins identified in *P. vivax* from patient. Interaction network of *P. vivax* proteins detected in our study has been constructed based on the presence of interactions of their *P. falciparum* homologs. The *P. vivax* proteins which have been detected in our study have been colored as red nodes. Many proteins detected in *P. vivax* from malaria patients form highly interconnected hubs showing the regulatory role of these proteins in several processes in vivax malaria. The major nodes have been indicated with an enlarged font and are hyperlinked to PlasmoDB.

In order to gain further insight into the functions of the *P. vivax* proteins detected in our study, we have constructed an interactome of the *P. vivax* proteome. For this, we first looked for *P. falciparum* homologs for the proteins detected in our study. We then selected those homologs for which interaction data was available in literature and found homologs for their interactors in the *P. vivax* genome [34], [35]. We then constructed an interactome consisting of *P. vivax* proteins detected in our study with other *P. vivax* proteins (Figure 3). Of the 153 proteins detected in our study, we found *P. falciparum* homologs for 137, 16 proteins were specific for *P. vivax*. Interactors for only 68 homologs could be found in literature and these were extrapolated to form the *P. vivax* interactome. In total, 309 interactions were included. Interestingly, many of the proteins found in our study form highly interconnected and busy networks in the parasite with unannotated hypothetical proteins indicating that these could be *Plasmodium*-specific pathways. For instance, MCM3, involved in chromosome maintenance, interacts directly with PVX_098620 and forms a network indirectly with SET domain containing protein and PVX_113635. Another highly interconnected hub is formed by Hsp86, nucleosome assembly protein 1 and Cg4, all of which have been detected in our study. Interestingly, Hsp86 is indirectly associated with Duffy receptor protein through Cg4 (member of Hsp70 family) indicating its involvement in virulence of the parasite. The *P. vivax* interactome for the proteins detected in our study indicate that highly interconnected and possibly active regulatory networks exist in the parasite, and several vivax-specific networks are awaiting discovery.

## Discussion

Research on human malaria caused by *P. vivax* has not received the attention that it deserves due to several factors. Firstly, unlike *P. falciparum*, *P. vivax* is not amenable to laboratory culture. Secondly (and more importantly), *P. vivax*, untill recently, was considered to be a parasite that causes mild and non-fatal disease. However, recent studies have shown that, in malaria endemic countries, like India, Indonesia, Papuea New Guinea, approximately 27% of the patients with severe malaria symptoms are due to P. vivax infection [36]. As with *P. falciparum*, drug resistance poses a severe problem in the treatment of *vivax*-malaria. Drug resistant strains of *P. vivax* have evolved against commonly used anti-malarials such as chloroquine, primaquine and sulphadoxine-pyrimethamine [37]–[39]. Notwithstanding the recent advances in global studies of *P. vivax* by transcriptomics and next generation sequencing [5], [6], [40], the amount of knowledge that exists about *P. vivax* is insufficient to understand its biology that will enable discovery of newer drug targets against the parasite.

Although transcriptome analysis documents a complete picture of the mRNA complement of a cell, they provide an inadequate view of the operational cellular protein networks. The absence of long term cultures of *P. vivax* both necessitates and provides us with an opportunity to study this parasite in the wild, i.e. directly from malaria-infected patients. In this context, it is essential to study the proteome of *P. vivax* in order to identify the molecular factors involved in *P. vivax* patho-biology. Proteomic analysis of the clinical isolates of *Plasmodium* is a difficult task owing to the low parasitemia and masking of parasite proteins by abundant host proteins.

Despite these technical shortcomings, we were able to isolate *P. vivax* parasites from patient's samples to enable a proteomic analysis. Although we could not completely eliminate host protein contamination, we were able to detect 153 *P. vivax* proteins in our study. Besides parasite proteins, we detected about 315 proteins of host-origin (Table S3). Of these, about 212 (Table S5) have been detected at transcript levels in the human reticulocyte transcriptome [41]. The other proteins may be from reticulocytes or remnants of other contaminating human blood cells such as leukocytes that have not been successfully removed from the sample.

**Figure 4:**
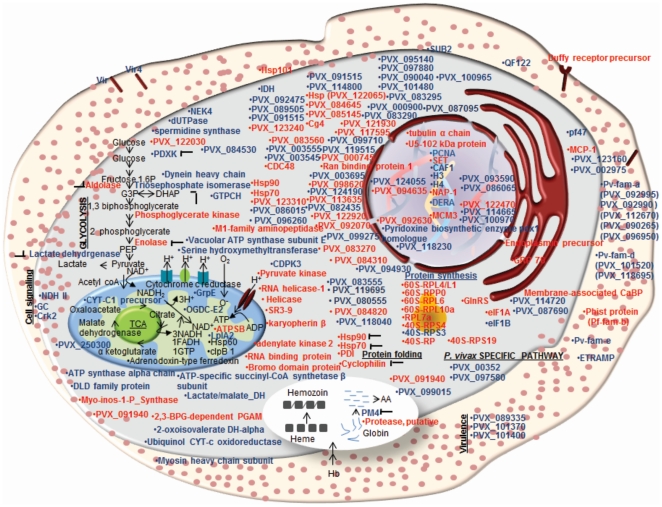
Schematic representations of pathways operational in the *P. vivax* from patient. The figure depicts the cellular localization of the identified proteins. Proteins indicated in blue represent the ones detected in the clinical isolate of *P. vivax* and black shows the pathways they are involved in. ‘
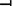
’ indicates potential targets for anti-malarials.

Additionally, 29 *P. vivax* proteins were detected for which no MS based evidence is available in any other *Plasmodium* species (Table S3). Majority (about 36%) of the detected *P. vivax* proteins consisted of hypothetical proteins. Further domain analysis by Pfam and SMART databases revealed putative function for 16 out of 55 hypothetical proteins detected in this study (Table S2). Amongst the 99 proteins with known function, enzymes involved in general metabolic functions were heavily represented (22 proteins), followed by proteins involved in chaperoning function (14) and translation (13) (Figure 2A). Several of these proteins are targets for chemotherapeutic interventions (Table 2). Notably, some of these proteins like PfA-M1, Spermidine synthase, Lactate dehydrogenase and hsp90 have been validated as drug targets. Besides some of the known drug targets, several proteins with immunogenic properties were detected which can be further exploited for designing vaccines. Most importantly, we found that the members of Pv-fam-a family (Tryptophan rich antigens) of proteins were abundantly expressed in the field isolates of *P.vivax* which warrants further evaluation of their candidature as vaccines. Figure 4 schematically describes the localization of the identified proteins in *P. vivax* and also gives a snap-shot of the biochemical pathways that would be operational in the clinical state of *P. vivax*.

We provide evidence for expression of four proteins which were hitherto not detected at the transcript level in earlier genome-wide analysis. This discrepancy can be explained in part due to an altered physiological state of the parasite in response to the host environment, though differential expression of genes by parasites from geographically distinct regions cannot be ruled out. The chief difference between the previous transcriptome analysis and this proteomics study lies in the regions of the world from where *P. vivax* samples have been collected. The two transcriptome analyses have collected samples from Thailand and Peru whereas our samples are from Mangalore, India. It is possible that different strains of *P. vivax* express marginally different protein complements.

An important finding of our study was the presence of 16 proteins that are entirely unshared by *P. falciparum* indicating that these proteins are likely involved in *P. vivax* specific infection. This is interesting considering the significant differences between *P. falciparum* and *P. vivax* infection such as preference for different host cells and extent of sequestration within host capillaries. It is possible that the unique proteins detected in our study are involved in important *vivax*-specific pathways.

This is the first proteomics analysis of *P. vivax*-an important first step towards understanding of *P. vivax* cell biology. Through this study, we have identified the most abundant proteins expressed in clinical *P. vivax* during its infection of the host such as possible regulatory proteins and strain specific proteins (Figure 4). These proteins provide a glimpse of the cellular pathways operational in the parasite. One limitation of this study is that, it was carried out with *P. vivax* samples pooled from different donors and hence we could not associate *in-vivo* parasite biological states with host environment, such as immune status of the patient. To better appreciate the host-parasite dynamics, additional cohort studies involving patients with asymptomatic parasitemia, mild and severe disease are required. This will not only facilitate identification of molecular factors important for clinical manifestation of malaria but will also lead to designing of better chemotherapeutic or vaccine intervention strategies.

## Supporting Information

Table S1 List of *P. vivax* proteins identified in clinical malaria sample and peptides identified.
(XLS)

Table S2 Functional classification of hypothetical proteins based on predicted PFAM domains.
(DOC)

Table S3 *P. vivax* proteins which do not have Mass Spectrometric evidence in any species of *Plasmodium*.
(XLS)

Table S4 Host proteins identified along with the proteome of *P. vivax*.
(XLS)

Table S5 *Plasmodium vivax* host proteins which are present at transcriptome level.
(XLS)

Table S6 *P. vivax* proteins detected in this study which have a score less than 0.47.
(XLS)
