# Supplementary material for: Clinical Proteomics of the Neglected Human Malarial Parasite Plasmodium vivax
Source: PLoS One. 2011 Oct 20;6(10):e26623. doi: 10.1371/journal.pone.0026623 (PMC3197670; doi:10.1371/journal.pone.0026623)
Supplement: Table S2 — Functional classification of hypothetical proteins based on predicted PFAM domains. (DOC) [file pone.0026623.s002.doc]

| **PlasmodbID** | **Description** | **Putative Function** | **Domains** |
| --- | --- | --- | --- |
| PVX_099275 | Hypothetical protein, conserved | DNA Repair | 1  668  136 264  407 553  ENDO 3  8-oxoguanine DNA glycosylase, N-terminal domain (136-264)  HhH-GPD superfamily base excision DNA repair protein (407-553) |
| PVX_092630 | Hypothetical protein, conserved | Chromatin Organization | 1  2712  45-103  Chr  Pfam: Chromodomain (45-103) |
| PVX_114665 | Hypothetical protein, conserved | Transcription initiation by RNA Pol II; part of core TFIIH complex,  Nucleotide excision repair | 1  322  20  302  Tfb 4  Pfam: Tfb 4 20-302 |
| PVX_084645 | Hypothetical protein, conserved | Translation | 1  667  362  554  eIF2A  Eukaryotic translation initiation factor eIF2A (362-554) |
| PVX_100970 | Hypothetical protein, conserved | RNA metabolism  1 | 2152  1207  1273  RRM  Pfam : RNA Recognition Motif (RRM): 1207-1273 |
| PVX_122470 | Hypothetical protein, conserved | RNA Processing;  RNA Metabolism; | 1152  1  859  1082  Pfam: MIF4G (**M**iddle domain of eukaryotic **i**nitiation **f**actor **4G** (eIF4G)) |
| PVX_124055 | Hypothetical protein, conserved | RNA Processing;  1  RNA Metabolism; | 13  58  190  330  S4  PseudoU_synth_2  409  S4 domain (13-58) RNA pseudouridylate synthase (190-330) |
| **PVX_087095** | Hypothetical protein, conserved | Cytoskeleton organization | 1  173  7  165  Profilin (7-165)  1485 - 1584 |
| PVX_094930 | Hypothetical protein, conserved | Signaling | 1  1642  443  562  SPRY  SPRY: 443-562; RanBPM-CRA: 1485-1584 |
| PVX_122030 | Hypothetical protein, conserved | Signal Transduction | **1**  8834  3136  2954  PI3Kc  Pfam: PI3K/PI4K 2954-3136 |
| PVX_123160 | Hypothetical protein, conserved | Serine type protease; Signal transduction; GTPbinding, GTPase | `  1  703  228  377  405  476  548  668  GTPase  PDZ  TRYPSIN  Pfam: Trypsin: 228 – 377; PDZ: 405 – 646; GTPase: 548 - 668 |
| PVX_084310 | Hypothetical protein, conserved | Protein-Protein interaction | 334  197  160  SHNi-TPR  1  SHNi-TPR; 160-197 |
| PVX_084820 | Hypothetical protein, conserved | Protein-Protein interaction | 559  7-40  328-271  313-345  373-406; 407-440; 441-474  TPR  TPR  TPR  TPR  TPR  TPR  1  TPR_1:- 7-40; 238-271; 313-345; 373-406; 407-440; 441-474. |

**Supplementary Table S2:** Functional classification of hypothetical proteins based on predicted PFAM domains. Column 1 and 2 contain Plasmodb ID’s and description, column 3 contains GO description of domains and column 4 contains a graphical illustration of domain arrangements (not to scale) in hypothetical proteins, domains are written according to their PFAM abbreviations alongside the encompassing amino acids for each domain. Hypothetical proteins were characterized on the basis of the presence of different domain viz. 8-oxoguanine DNA glycosylase, HhH-GPD superfamily base excision DNA repair protein, chromodomain, Transcription factor 4b, Eukaryotic translation initiation factor eIF2A, RNA Recognition Motif (RRM), **M**iddle domain of eukaryotic **i**nitiation **f**actor **4G** (MIF4G), Profilin, S4 domain , RanBPM-CRA SPRY (SPRY Domain is named from SPla and the RYanodine Receptor.), RNA pseudouridylate synthase, PI3K (Phosphoinositide 3-kinase ), Trypsin, PDZ, GTPase, and TPR (Tetratricopeptide repeat).
